# Mechanistic Insights of the Interaction of Plant Growth-Promoting Rhizobacteria (PGPR) With Plant Roots Toward Enhancing Plant Productivity by Alleviating Salinity Stress

**DOI:** 10.3389/fmicb.2020.01952

**Published:** 2020-08-20

**Authors:** Mujtaba Aamir Bhat, Vijay Kumar, Mudasir Ahmad Bhat, Ishfaq Ahmad Wani, Farhana Latief Dar, Iqra Farooq, Farha Bhatti, Rubina Koser, Safikur Rahman, Arif Tasleem Jan

**Affiliations:** ^1^Department of Botany, School of Biosciences and Biotechnology, Baba Ghulam Shah Badshah University, Rajouri, India; ^2^Department of Biotechnology, Yeungnam University, Gyeongsan, South Korea; ^3^Department of Biotechnology, School of Biosciences and Biotechnology, Baba Ghulam Shah Badshah University, Rajouri, India; ^4^Department of Microbiology, School of Biosciences and Biotechnology, Baba Ghulam Shah Badshah University, Rajouri, India; ^5^Department of Botany, Munshi Singh College, Babasaheb Bhimrao Ambedkar Bihar University, Muzaffarpur, India

**Keywords:** plant productivity, phytohormones, rhizosphere, PGPR, soil salinity

## Abstract

Agriculture plays an important role in a country’s economy. The sector is challenged by many stresses, which led to huge loss in plant productivity worldwide. The ever-increasing population, rapid urbanization with shrinking agricultural lands, dramatic change in climatic conditions, and extensive use of agrochemicals in agricultural practices that caused environmental disturbances confront mankind of escalating problems of food security and sustainability in agriculture. Escalating environmental problems and global hunger have led to the development and adoption of genetic engineering and other conventional plant breeding approaches in developing stress-tolerant varieties of crops. However, these approaches have drawn flaws in their adoption as the process of generating tolerant varieties takes months to years in bringing the technology from the lab to the field. Under such scenario, sustainable and climate-smart agricultural practices that avail bacterial usage open the avenues in fulfilling the incessant demand for food for the global population. Ensuring stability on economic fronts, bacteria minimizes plant salt uptake by trapping ions in their exopolysaccharide matrix besides checking the expression of Na^+^/H^+^ and high-affinity potassium transporters. Herein we describe information on salinity stress and its effect on plant health as well as strategies adopted by plant growth-promoting rhizobacteria (PGPR) in helping plants to overcome salinity stress and in mitigating loss in overall plant productivity. It is believed that acquisition of advanced knowledge of plant-beneficial PGPR will help in devising strategies for sustainable, environment-friendly, and climate-smart agricultural technologies for adoption in agriculture to overcome the constrained environmental conditions.

## Introduction

With rapid urbanization, the reduction in agricultural land left less space to expand the cultivation of plants. Under such circumstances, expansion in plant production relies on increasing the fertility of soils to ensure food for all under the current global food security scenario (Godfray et al., 2010). In this direction, soil quality and water availability play a pivotal role in sustainable agricultural productivity. Any disbalance of salt in soil and water leads not only to decline in plant productivity but also even to their abandonment as it progresses with change in the land pattern from fertile to a marginal one. Although primary salinity is natural in the environment, the contribution by anthropogenic sources such as urbanization and deforestation is worth noting as these result in enhancing loss of the cultivable capacity of soils (land degradation and disturbance in the physical and the biological properties of soil) that affect plant productivity worldwide. Enhancement in salt deposits in an agricultural field hampers the growth of crop plants. In the scenario of decreased availability of fertile land, studies were directed in adopting genetic engineering approaches to complement traditional breeding methods in the development of salt-tolerant crops of food and fiber (Rozema and Flowers, 2008; Zhu et al., 2011; Dodd and Perez-Alfocea, 2012; Joshi et al., 2015). Despite significant efforts, the complexity in understanding the biological aspects of salt-stress-induced changes (morphological, biochemical, and physiological) renders limited success in developing salinity-stress-tolerant plants.

To cope up with the limited success in bringing technology-driven transgenics from the lab to the field, alternative strategies, such as the introduction of salt-tolerant microbes, are explored for adoption in augmenting and, as such, enhancing the growth of crops in salt-affected soils (Dodd and Perez-Alfocea, 2012; Etesami and Beattie, 2017; Etesami, 2018). Among them, plant growth-promoting rhizobacteria (PGPR) constitutes an important class of microorganisms that were found effective in inducing systemic tolerance in plants to tolerate abiotic stresses (Dutta and Khurana, 2015; Etesami and Beattie, 2018). However, PGPR from hypersaline soils (halotolerant PGPR) expressing plant growth-promoting (PGP) traits were found least affected by environmental factors such as climate, soil characteristics, etc., and thus are more efficient in enhancing salt tolerance in plants than PGPR from non-saline habitats (Giongo et al., 2008; Upadhyay et al., 2009; Egamberdieva and Kucharova, 2009; Khan et al., 2016). As part of the plant–bacterial interaction at the rhizospheric plane, plants were found to dictate the growth of microbiota for driving adaptation to changing environmental conditions (Berendsen et al., 2012). While many excellent reviews discussed a range of diverse plant-beneficial traits of microbiota encompassing both bacteria and fungi (Qin et al., 2016; Ilangumaran and Smith, 2017; Etesami and Beattie, 2017, 2018; Backer et al., 2018; Egamberdieva et al., 2019), the present study is aimed at highlighting the importance of plant–bacterial interactions, with comprehensive inputs about the mechanistic insights that operate at the plant level in mitigating salt stress toward improvement in crop yield as part of the climate-smart agricultural practices geared for feeding the ever-increasing global population.

## Soil Salinity and Plant Growth

Increase in the concentration of salts, preferably sodium chloride (NaCl; electrical conductance >4 dSm^–1^ or 40 mM), attributed to both natural (salts released by weathering of rocks, salt from seawater influx, air-borne salts from oceans, etc.) and anthropogenic (surface runoff and irrigation-based salt deposition year after year) sources, renders the soil no longer suitable for cultivation (Pitman and Lauchli, 2002; Rengasamy, 2002). Despite suitable soil water columns, excessive salinity raising the concentration in soil solutions deprive plants of using it *via* osmotic reduction. High soil salt concentrations induce its effects right from imbibition of water to seed germination and root elongation that together have a great effect on the yield of crop plants (Katembe et al., 1998; Kaymakanova, 2009). It has been observed that the pre-treatment of seeds with different PGPR promotes seed germination and seedling growth (Poupin et al., 2013; Rahmoune et al., 2017; Bakhshandeh et al., 2020). As part of the mechanism, it is believed that PGPR helps in maintaining the balance of hormones, e.g., auxin to cytokinin levels during germination and the early stages of plant development, thereby playing a critical role in dictating the genetic program that controls post-embryonic roots and shoot growth (Chu et al., 2019; Qessaoui et al., 2019).

At later stages of plant growth, soil salinity interferes with root turgor that led to reduction in water absorption, decrease in the plant water column that progresses through dehydration and osmotic stress, inhibition of the metabolic machinery, disturbance in the transpiration system, and, most importantly, interference with parameters pertaining to photosynthesis (Kaushal and Wani, 2015). Photosynthesis refers to a major attribute in dry matter and, as such, in plant productivity, showing a decrease in saline condition owing to the reduction in leaf turgor and reduced leaf surface area (Qin et al., 2010; Tanveer and Shah, 2017). It occurs either through (1) decreased stomatal opening and CO_2_ uptake, which in turn is associated with the reduction in stomatal conductance or (2) operation of a less-efficient Calvin cycle due to limited chlorophyll content (Lycoskoufs et al., 2005; Chaves et al., 2009). Stunted growth (seedling) with reduced biomass and leaf area are observed effects of salinity stress in the growth (vegetative stage) of plants (Takemura et al., 2000; Wang et al., 2003). PGPR employ different mechanisms in encouraging plant growth, prominently being nutrient availability and securing mineral assets such as phosphorus, phytohormone production, production of volatile compounds in controlling seed- and soil-borne phytopathogen, and synergism with other plant-beneficial microorganisms in enhancing resistance against different stresses (Bhattacharyya and Jha, 2012; Bhattacharyya et al., 2015; Bell et al., 2015; Kurepin et al., 2015; Bach et al., 2016; Yuan et al., 2016). Additionally, a limited canopy that prevents water loss by transpiration also constitutes a plant survival mechanism under high salt concentrations (Savé et al., 1994; Ruiz-Sánchez et al., 2000; Colmer et al., 2005; Cassaniti et al., 2009, 2012).

## Salinity Stress, PGPR, and Plant Productivity: A Triangular Conjecture

Salinity is a stress of global magnitude, having a substantial effect on plant growth, and is accountable for a significant loss in their productivity. Exerting adverse effects on germination, vigor, and yield, it led to drastic reduction in plant productivity, as observed in plants growing in arid and semi-arid areas (Paul and Lade, 2014). With an increase in salt concentration, disturbance in the cellular ion balance led to an enhancement in reactive oxygen species (ROS) production, besides taking a huge toll in exerting ionic toxicity on the accumulation of Na^+^ and Cl^–^ ions (Grover et al., 2011). ROS (free oxygen radicals, superoxide, and hydrogen peroxide) are capable of damaging cellular structures and damage of biomolecules (proteins, lipids, etc.) besides talking a huge toll on chlorophyll degradation and lipid peroxidation that are, in turn, associated with a reduction in photosynthetic activity, damage of cellular membranes, and ultimately proceeding with induction of programmed cell death (Apel and Hirt, 2004). Interfering in cellular enzymatic functions, the accumulation of Na^+^ and Cl^–^ ions produces diverse effects on different physiological fronts and in its effect on the growth and the development of plants (Nunkaew et al., 2015; Acosta-Motos et al., 2017). Photosynthesis capacity is reduced due to the interference of these ions with the opening and the closing of the stomata and in exerting osmotic stress as reflected in plants through reduction in leaf area and chlorophyll content (Munns, 1992; Kang et al., 2014a). Suppression of plant growth, a phenomenon of disturbed metabolic activities as a result of nutritional and hormonal imbalance together with abscission and senescence, is observed once the intensity of salinity stress, together with temperature, crosses the limit (Glick, 2014; Paul and Lade, 2014; Hashem et al., 2015). The accumulation of Cl^–^ ion leads to inhibition of nitrate reductase activity in the photosynthetic pathway (Azza Mazher et al., 2007; Nadeem et al., 2014). Elevation in ethylene (C_2_H_4_) levels progresses with drastic effects on plant health such as defoliation, senescence, etc. (Barnawal et al., 2014; Glick, 2014). Upon overcoming the storage capacity of cells, the accumulation of salts progresses to dehydration of cells, ultimately leading to plant death (Kang et al., 2014a).

**FIGURE 1:**
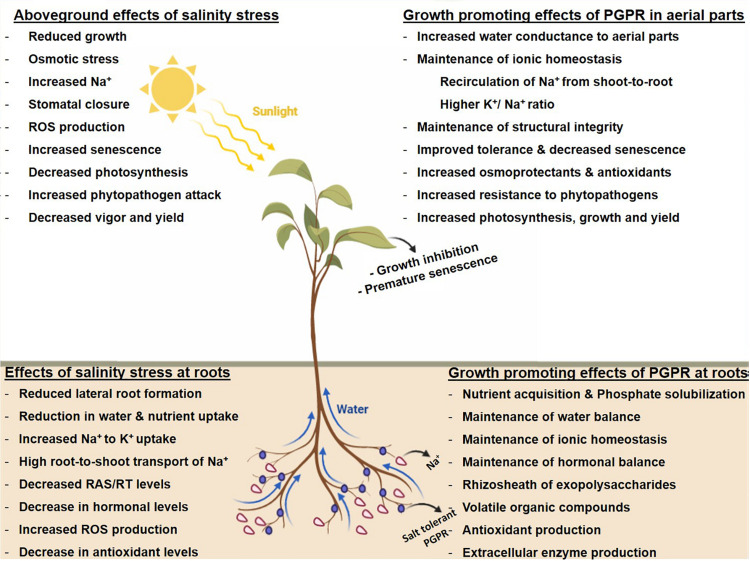
Salinity stress and tolerance mechanisms induced by plant growth-promoting rhizobacteria.

Constituting an excellent environment for them to flourish, plant-beneficial microorganisms play an important role in achieving sustainability in plant productivity under the current paradigm of climatic change. As part of the climate-smart agricultural practices, microorganisms improve nutrient availability to plants and, in return, get nutrients as root exudates from these plants (Patel et al., 2015; Hamilton et al., 2016; Singh and Strong, 2016). Halotolerant PGPR employs a wide range of strategies as adaption for survival under saline conditions and, in turn, executes a number of plant-beneficial mechanisms for improving the growth of crop plants growing under salinity stress (Figure 1). These include (1) making nutrients available to plants *via* solubilization of phosphorus and potassium, siderophore production for iron uptake, and fixation of atmospheric nitrogen (Etesami and Beattie, 2017; Etesami, 2018), (2) maintenance of water balance by changing the architecture of roots for hydraulic conductance (Arora et al., 2012), (3) selective uptake of K^+^ to Na^+^ ions for maintaining a high K^+^/Na^+^ ratio that indirectly reduces the intercellular accumulation of K^+^ to Na^+^ ions (Islam et al., 2016; Etesami, 2018), (4) exopolysaccharide (EPS)-mediated alleviation of salt stress by decreasing Na^+^ accumulation in roots and, as such, preventing their translocation to the leaves (Nunkaew et al., 2015; Qin et al., 2016; Etesami and Beattie, 2017), (5) production of volatile compounds and osmoprotectants that enhance the plants’ survival under salt stress (Creus et al., 2004; Timmusk et al., 2014), (6) protecting plants from oxidative stress by upregulating the activity of enzymes such as superoxide dismutase (SOD), catalase (CAT), and peroxidase as part of the antioxidant defense system (Islam et al., 2016), (7) maintenance of hormonal level for alleviation of salt stress (Etesami et al., 2014; Singh et al., 2015; Etesami and Beattie, 2017), (8) modulation in the expression of stress-responsive genes (Gond et al., 2015; Qin et al., 2016; Kaushal and Wani, 2016; Etesami and Beattie, 2017), and (9) production of extracellular enzymes that impart protection against phytopathogens competing with beneficial bacterial species for nutrients (Hariprasad et al., 2011; Dubey et al., 2014; Etesami, 2018).

## PGPR in the Alleviation of Salt Stress

Salinity stress adversely affects plant morphological, physiological, and biochemical functioning that, in turn, proves detrimental to plant health. Salt tolerance−a parameter quantified over given time−is survival, growth (vegetative), and biomass (harvestable) of the plant growing under salt stress to non-saline habitats (Munns, 2002b). The plants adopt either by inheriting genetic traits that impart salinity tolerance or by adopting a selectable mechanism of salt exclusion from the roots, thereby delaying salinity stress (Munns, 2002b; Zhu, 2007). A few (in particular, halophytes) conduct movement of accumulated salts *via* the xylem for precipitation at the leaf surface, while others have developed specialized structures (salt glands) in shoots, whereby salt is excreted on the surface for removal by wind or water (Ilangumaran and Smith, 2017). Additionally, plants undergo valuable interactions with bacterial species residing in the rhizospheric region, with an interaction pattern ranging from mutualism to antagonism. Colonization and successful establishment in the rhizospheric region are considered as a prerequisite for their interaction at the root surface. Traits that promote colonization of PGPR at the root surface include the availability of sufficient nutrients besides the property of being motile and capable of adherence (*via* pilli, surface-localized proteins, etc.) to plant roots (Jan et al., 2011). On one side where root exudates (organic acids, phenolics, sugars, amino acids, etc.) help microbes to flourish, it prompts changes (both physical and chemical) in plants related to defense, nutrient deficiency, and tolerance against heavy metals besides being important in eliciting strong responses against different abiotic stresses such as salinity as a mechanism of promoting plant growth (Jan et al., 2011; Nadeem et al., 2014; Rashid et al., 2016). Table 1 a detailed account of the growth-promoting attributes of PGPR in agroecosystems is given in the following discussion.

**TABLE 1:** Plant growth-promoting rhizobacteria (PGPR)−plant interactions under salinity stress and plant beneficial effects recorded thereof.

**Sample number**	**Plant species**	**PGPR species inoculation**	**Effects observed**	**References**
(1)	Maize (*Zea mays*)	*Achromobacter xylosoxidans*	Improved maize growth and productivity under drought stress	Danish et al., 2020
		*B. licheniformis* FMCH001	Enhances plant water use efficiency *via* growth stimulation in both normal as well as in drought conditions	Akhtar et al., 2020
		*Bacillus* sps.	Induces plant response for defense enzymes, chlorophyll, proline, and soluble sugar under salt stress	Misra and Chauhan, 2020
		*Bacillus* sp. NBRI YN4.4	Improves photosynthetic pigments and soluble sugar content and decreases proline level under stress conditions; also enhances soil enzymes dehydrogenase, alkaline phosphatase, and betaglucosidase, which help in improving soil health	Dixit et al., 2020
		*Ochrobactrum* sp. NBRISH6	Helps in maintaining homeostasis through various mechanisms under deficit water stress condition	Mishra et al., 2020
		*A. brasilense*	Induced the development of a more extensive root system, regardless of growth medium nitrate concentration	Pii et al., 2019
		*Burkholderia cenocepacia* CR318	Helps in the health and the growth of crop including phosphate and potassium solubilization and antimicrobial activity	You et al., 2020
		*P. aeruginosa* strain FB2 and *B. subtilis* strain RMB5	Shows effectivity against a range of fungal phytopathogens	Ali et al., 2020
		*Serratia liquefaciens* KM4	Maintenance of water balance, enhanced antioxidant enzyme activities, increased nutrient uptake	El-Esawi et al., 2018b
		*Pseudomonas* sp., *Arthrobacter* sp., *Bacillus* sp., and members of other bacterial groups	Enhanced phosphate solubilization, IAA and ACC deaminase activity	Aslam and Ali, 2018
		*A. brasilense* Ab-V5 and Ab-V6, *Rhizobium tropici* CIAT 899	Enhanced antioxidant enzyme activities	Fukami et al., 2018
		*Bacillus aquimaris* DY-3	Maintenance of water balance, development of pigment system, enhanced antioxidant enzyme activities	Li and Jiang, 2017
		*Bacillus amyloliquefaciens* SQR9	Enhanced solute accumulation, enhanced antioxidant enzyme activities, increased expression of salinity stress response genes	Chen et al., 2016
		*Staphylococcus sciuri*	Enhanced antioxidant enzyme activities	Akram et al., 2016
		*Bacillus* spp., *Arthrobacter pascens*	Phosphate solubilization, maintenance of water balance, increased antioxidant enzyme activities	Ullah and Bano, 2015
		*Pantoea agglomerans*	Increased expression of aquaporin genes	Gond et al., 2015
		*P. syringae*, *P. fluorescens*	Enhanced ACC deaminase activity	Zafar-ul-Hye et al., 2014
		*Proteus penneri*, *P. aeruginosa*, *A. faecalis*	Enhanced exopolysaccharide production	Naseem and Bano, 2014
		*Azotobacter chroococcum*	Enhanced growth, increased phosphate solubilization and K^+^/Na^+^ ratio	Rojas-Tapias et al., 2012
		*Bacillus megaterium*	Improved expression of ZmPIP isoforms	Marulanda et al., 2010
		*Rhizobium*, *Pseudomonas* spp.	Osmotic regulation	Bano and Fatima, 2009
		*Pseudomonas* spp., *Enterobacter* spp.	ACC deaminase activity	Nadeem et al., 2009
		*Pseudomonas syringae*, *Enterobacter aerogenes*, *P. fluorescens*	ACC deaminase activity	Nadeem et al., 2007
		*Azospirillum brasilense*	Maintenance of ion homeostasis, decreased nitrogenase activity	Hamdia et al., 2004
(2)	Rice (*Oryza sativa*)	*Bacillus aryabhattai*, *Achromobacter denitrificans*, and *Ochrobactrum intermedium*	Helps to accumulate under salt stress and exhibits greater resistance to heavy metals	Sultana et al., 2020
		*Klebsiella* sp. PD3	Degrades phenanthrene; also shows ACC deaminase activity and phosphate solubilization	Li X. et al., 2020
		*Bacillus amyloliquefaciens* SN13	Induces metabolic and physiological parameters *via* different enzymes to reduce the impact of stress	Bisht et al., 2019
		*Bacillus* sp. JBS-28	Promotes grain yields; also decreases arsenic accumulation in arsenic-contaminated soil and paddy fields	Aw et al., 2019
		*Bacillus aryabhattai* MS3	Phosphate solubilization, enhanced siderophore and IAA production	Sultana et al., 2018
		*Halobacillus dabanensis* SB-26, *Halobacillus* sp. GSP 34	Nitrogen fixation and IAA production	Rima et al., 2018
		*Enterobacter* sp. P23	Growth promotion, phosphate solubilization, increased siderophore, and IAA production, reduction in ethylene production, enhanced antioxidant enzyme activities	Sarkar et al., 2018
		*B. stratosphericus* (NBRI 5Q and NBRI 7A)	Increased growth and biomass production, Phosphate solubilization, IAA production, enhanced ACC deaminase activity	Misra et al., 2017
		*Thalassobacillus denorans* (NCCP-58), *Oceanobacillus kapialis* (NCCP-76)	Increased germination and growth of root and shoot, developed pigment system, reduced Na^+^ ion accumulation	Shah et al., 2017
		*Bacillus pumilus*	Growth promotion, enhanced antioxidant enzyme production, reduced Na^+^ ion accumulation	Khan et al., 2016
		*Bacillus* and *Citrobacter*	Growth promotion, phosphate solubilization, IAA production	Habib et al., 2016
		*Pseudomonas* PF1 and TDK1	Enhanced antioxidant enzyme production	Sen and Chandrasekhar, 2015
		*Serratia* sp., *Pseudomonas* sp.	Growth promotion, phosphate solubilization, IAA production	Nakbanpote et al., 2014
		*Alcaligens* sp., *Bacillus* sp., *Ochrobactrum* sp.	ACC deaminase activity	Bal et al., 2013
		*P. pseudoalcaligenes, B. pumilus*	Reduction in ROS production, delay of senescence	Jha and Subramanian, 2013
		*B. amyloliquefaciens* NBRISN13 (SN13)	Solute accumulation, enhanced expression of SOS1, EREBP, SERK1, and NADP-Me2	Nautiyal et al., 2013
(3)	Wheat (*Triticum aestivum*)	*Variovorax paradoxus* RAA3; *Pseudomonas* spp. DPC12, DPB13, DPB15, DPB16; *Achromobacter* spp. PSA7, PSB8; *Ochrobactrum anthropi* DPC9	ACC deaminase activity; improves the growth of plants in water-stressed rain-fed environments	Chandra et al., 2019
		*Planomicrobium chinense and Bacillus cereus* with salicylic acid	Reduces moisture stress in plants	Khan and Bano, 2019
		*Bacillus siamensis, Bacillus* sp., and *Bacillus methylotrophicus*	ACC deaminase activity	Amna et al., 2019
		*Bacillus subtilis*	Induction of systemic resistance	Lastochkina et al., 2017
		*Dietzia natronolimnaea*	Enhanced expression of SOS-related genes, increased tissue-specific expression of ion transporters, modulation of ABA signaling cascade	Bharti et al., 2016
		*Serratia marcescens* CDP-13	ACC deaminase activity, minimizes the salinity-induced oxidative damages to the plants	Singh and Jha, 2016
		Arthrobacter spp. SU18, *B. aquimaris* SU44, *B. aquimaris* SU8	Root dry weight and shoot biomass	Upadhyay and Singh, 2015
		*Azosprillium lipoferum*, *Pseudomonas fluorescens* 169	Development of pigment system	Saghafi et al., 2013
		*Azospirillum*	Development of pigment system, enhanced solute accumulation, increased seedling growth and plant yield	Nia et al., 2012
		*Piriformo sporaindica, Azospirillum*	Development of pigment system, enhanced solute accumulation, increased seedling growth	Zarea et al., 2012
		*Azospirillum lipoferum*	Growth and biomass accumulation	Bacilio et al., 2004
(4)	Soybean (*Glycine max*)	*Bradyrhizobium diazoefficiens* USDA110, *Bacillus velezensis* S141	Enhanced nodulation and N2-fixing efficiency by producing larger nodules	Sibponkrung et al., 2020
		*Bradyrhizobium*	Improves plant development and increases nodulation	Zeffa et al., 2020
		*P. fluorescens* LBUM677	Enhances plant biomass, oil content, and lipid composition	Jiménez et al., 2020
		*A. woluwensis, M. oxydans, A. aurescens, B. megaterium*, and *B. aryabhattai*	Maintains osmotic balance and regulates salt tolerance	Khan et al., 2019
		*L. adecarcoxylata* LSE-1, *Bradyrhizobium* sp. LSBR-3	Promotes plant growth with increased plant productivity	Kumawat et al., 2019
		*Bacillus firmus* SW5	Development of root system, enhanced antioxidant enzyme levels	El-Esawi et al., 2018a
		*Bradyrhizobium japonicum* USDA 110, *P. putida* TSAU1	Development of root system with nodule formation, increased phosphate acquisition	Egamberdieva et al., 2017
		*Pseudomonas simiae* AU	Increased chlorophyll content, phosphate solubilization, IAA and siderophore production; decrease in Na^+^ accumulation at root surface	Vaishnav et al., 2016a
		*Bacillus thuriengenesis* NEB17	Increased PEPCO and RuBisCo expression, enhanced production of pyruvate kinase, proteins of photosystems I and II, isocitrate lyase, and antioxidant glutathione-S-transferase	Subramanian et al., 2016
		*P. putida* H-2-3	Enhanced production of ABA, salicylic acid, and gibberellins	Kang et al., 2014b
		*P. fluorescens*	Enhanced cytokinin production	Bhattacharyya and Jha, 2012
		*Bradyrhizobium japonicum*, *Bacillus subtilis* SU-12, *Serratia proteamaculans*	Exopolysaccharide production, antioxidant activity	Han and Lee, 2005
(5)	Tomato (*Solanum lycopersicum*)	*Bacillus subtilis* Rhizo SF 48	ACC deaminase activity; protects against oxidative damage and enhances plant growth against drought stress	Gowtham et al., 2020
		*Funneliformis mosseae*, *Enterobacter* sp. EG16, and *Enterobacter ludwigii* DJ3	Enhances plant growth and tolerance to Cd in Cd-contaminated soil	Li Y. et al., 2020
		*Leclercia adecarboxylata* MO1	IAA- and ACC-deaminase-producing abilities; improves plant tolerance to salinity stress	Kang et al., 2019
		*Pseudomonas putida* UW4 (ACC deaminase)	Increased shoot growth and expression of Toc GTPase	Yan et al., 2014
		*Pseudomonas aeruginosa* T15, *Pseudomonas fluorescens* NT1, *Pseudomonas stutzeri* C4	Decreased ethylene levels, increased root and shoot length	Tank and Saraf, 2010
		*Achromobacter piechaudii* ARV8	Enhanced induced systemic tolerance, enhanced ACC deaminase activity	Mayak et al., 2004
(6)	Common bean (*Phaseolus vulgaris*)	*Aneurinibacillus aneurinilyticus* and *Paenibacillus* sp.	ACC deaminase activity	Gupta and Pandey, 2019
		*Mycorrhizae*, *Bacillus subtilis*, and *Pseudomonas fluorescence*	Controls the infection of *Sclerotium rolfsii*; also acts as biofertilizers	Mohamed et al., 2019
		*Rhizobium*	Increased nutrient content and dry weight	Yanni et al., 2016
		*Pseudomonas chlororaphis* TSAU13, *Pseudomonas extremorientalis* TSAU20	Increased dry weight and root length	Egamberdieva, 2011
		*Azospirillum brasilense*, *Rhizobium* spp.	Enhanced root branching, increased secretion of flavonoids	Dardanelli et al., 2008
(7)	Radish (*Raphanus sativus*)	*Bacillus* sp. CIK-516	Improves plant growth and enhances Ni phytoextraction	Akhtar et al., 2018
		*Lactobacillus* sp., *P. putida* and *Azotobacter chroococcum*	Helps to mitigate salinity stress at the time of germination	Hussein and Joo, 2018
		*Arthrobacter scleromae* SYE-3	Increased shoot length	Hong and Lee, 2017
		*Staphylococcus kloosii*, *Kocuria erythromyxa*	Increased chlorophyll content, increased shoot and root fresh and dry weight	Yildirim et al., 2008a
		*Bacillus* spp.	Induction of plant growth	Yildirim et al., 2008b
(8)	Barley (*Hordeum vulgare*)	*Hartmannibacter diazotrophicus*	Growth induction, enhanced ACC deaminase activity, increased root and shoot dry weight	Suarez et al., 2015
		*Curtobacterium flaccumfaciens*	Promotes plant growth	Cardinale et al., 2015

### Maintenance of Water Balance and Nutrient Acquisition

The hydration of cells, having a greater impact on physiological and metabolic processes, determines behavioral growth in plants. Hydraulic gradients in the xylem regulate water conductance from the roots to the leaves against an imbalance between the rate of transpiration and the available water absorbed from the soil (Passioura, 2010; Chavarria and dos Santos, 2012). The sustained transpiration of water from the leaf surface without any replenishment causes a reduction in xylem water potential that progressively leads to leaf dehydration, depending on the environmental conditions, stage of the growth of plant, canopy characteristics, and water quality as part of irrigation. The accumulation of salts at the root surface causes a transition in the root architecture (supresses lateral root formation) over time that influences the availability and uptake of soil nutrients. Salinity-induced osmotic stress proceeds with a decrease in diffusion and, as such, mass flow of nutrients as they are carried to the roots of plants by water (Zhu, 2001; Munns, 2002a; Ashraf, 2004; Sánchez-Blanco et al., 2004; Meloni et al., 2008; Franco et al., 2011; Chavarria and dos Santos, 2012). Under osmotic stress conditions, the aboveground plant parts undergo little photosynthetic activity and switch to the use of photo-assimilates, which causes a reduction in plant growth. All these events lead to a subsequent reduction in plant productivity (Chartzoulakis et al., 2002; Giri et al., 2003; Katerji et al., 2005; Bhatnagar-Mathur et al., 2007; Álvarez et al., 2012; Gómez-Bellot et al., 2013).

The inoculation of bacterial isolates to the roots of pepper plants resulted in an enhanced roots system, thereby increasing the ability of plants to uptake water from the surroundings (Marasco et al., 2013). The expression of aquaporins (water-conducting proteins) present in plasma and intracellular membrane determines the hydraulic conductance (*L*) at the root surface and, as such, the uptake of water from salinized soil for a plant (Moshelion et al., 2015; Qin et al., 2016). Plasma membrane intrinsic proteins (*PIP*s) constitute important aquaporins for a plant, which helps in its adaptation to changing environmental conditions (Marulanda et al., 2010; Moshelion et al., 2015). An expressional analysis of *Zea mays* roots inoculated with *Bacillus megaterium* and *Pantoea agglomerans* showed up-regulated *PIP2* and *ZmPIP1-1* genes that contribute to the increase in the *L*-values under salinity stress conditions (Gond et al., 2015). These studies reveal that PGP bacteria determine the resistance of plants to water stress irrespective of the nature of interaction in determining the specificity for growth-promoting activity. Plant−bacterial interactions at the root surface assist plants in maintaining the availability of water and helps in the acquisition of nutrients through nitrogen fixation, phosphate solubilizations, and siderophore production as part of their mechanism in fulfilling the nutritional requirements of plants (Beattie, 2015; Pii et al., 2015). Nitrogen−an essential nutrient that limits plant productivity−is often applied exogenously. However, inorganic fertilizers that compensate nitrogen deficiency often lead to a change in soil structure and, as such, composition of soil microflora (Rueda-Puente et al., 2003). Studies were performed on exploring the naturally occurring nitrogen fixers which have the potential for exploration toward plant growth promotion. Of the different interactions, the nitrogen-fixing assembly of rhizobia in the roots of legumes is an extensively studied relationship between plants and bacteria. In this symbiotic relationship, the rhizomes provide the legumes with nitrogen and, in return, get reduced carbon as nutrient and suitable environment for nitrogenase activity (Backer et al., 2018). Being a sensitive process, all stages of nitrogen fixation in leguminous plants were found to be prone to salinity effects, which result in a decrease in the nitrogen content of leguminous plants (de la Peña and Pueyo, 2012; Bruning and Rozema, 2013). In this regard, the commercial preparation of halotolerant free-living diazotrophs such as *Azotobacter* sp., *Azospirillium* sp., etc., proved beneficial than rhizobia in nitrogen fixation in a variety of crops worldwide, thereby found effective in increasing the yield of various cereal crops (Vessey, 2003; Bashan and de-Bashan, 2015; Sharma et al., 2016).

Phosphorus is a major essential macronutrient that constitutes another limiting nutrient for plants after nitrogen. The abundance of insoluble forms and the intensive agricultural practices in both saline and fertile soils deplete plants of this essential nutrient. On the second line, phosphate-solubilizing microorganisms (PSMs) convert and as such make non-soluble forms of phosphate to easily available soluble forms for efficient utilization by the plants (Backer et al., 2018). Compared to complementation with NPK fertilizers, the employment of phosphate-solubilizing bacteria was found effective in enhancing phosphate availability to plants without exacerbating the soil salinity levels (Etesami, 2018; Etesami and Beattie, 2018). The liberation of reactive forms of phosphate from organic compounds on utilizing enzyme phytase of PSMs constitutes another mode of phosphate availability to plants. Additionally, the production of hydrogen cyanide (HCN), which was earlier thought as a plant-protective mechanism, was found to be associated with an enhancement in phosphate availability to plants (Rijavec and Lapanje, 2016). Siderophore (iron-binding ligands) production is associated with the deprivation of pathogenic microorganisms of iron (a micronutrient) and making it available for use in respiration, photosynthesis, and nitrogen fixation by plants (Ahmed and Holmstrom, 2014; Saha et al., 2016).

### Maintenance of Ionic Homeostasis

Alleviating the nutritional imbalance caused by a high influx of salt ions regulates the exchange of nutrients (both macro and micro) to minerals. Microbes increase nutrient availability to plants through the increased production of siderophores (metal chelation) and bringing changes in pH at the surface of rhizospheres (Dodd and Perez-Alfocea, 2012; Lugtenberg et al., 2013). Disturbance in ionic homeostasis is observed in crops that are poor excluders of Na^+^ (rice, beans, etc.) and sensitive to Cl^–^ ions (citrus, soybean, etc.) grown in soils with high salt levels (Munns, 2002a; Tester and Davenport, 2003). Under salinity stress, the influx of Na^+^ into the roots undergoes translocation to the aerial parts *via* the xylem, with the final accumulation taking place at the leaf surface rather than at the roots (Tester and Davenport, 2003). As such, excluding Na^+^ from plants becomes difficult as only a small proportion of it undergoes recirculation to the roots *via* the phloem, thereby restricting it to the aerial parts, thus causing toxicity in plants. An increase in the concentration of Na^+^ disturbs the Na^+^/K^+^ ratio that progresses with the inhibition of cytosolic activities besides interfering with the activities of enzymes involved in respiration and photosynthesis (Baral et al., 2015; Jacoby et al., 2016). Considering the importance of Na^+^ homeostasis to the growth of plants, the regulatory network of Na^+^/H^+^ antiporter and high-affinity K^+^ transporters (HKT) is put to work for the efflux of Na^+^ ions from the cells throughout the plants (Tester and Davenport, 2003; Davenport et al., 2005). With localization on the plasma membrane, the Na^+^/H^+^ antiporter (also referred to as SOS1, salt overlay sensitive channel) efflux Na^+^ in response to its increasing cytosolic levels (Qiu et al., 2002). Also, the increase in plant Na^+^ level interferes with the uptake of K^+^ at the root surface *via* the low-affinity K^+^ uptake system. To increase salinity tolerance, plants activate high-affinity K^+^ transporters, thereby increasing the uptake of K^+^ over Na^+^ ions in plants (Rodríguez-Navarro and Rubio, 2006). Additionally, the activation of membrane-bound Ca^2+^ channels in response to a depolarization event generates a Ca^2+^ signal that indicates the occurrence of salt stress in plants. The Ca^2+^ signal is sensed by calcineurin B-like protein (CBL4; also referred to as SOS3) which undergoes complex formation with CBL-interacting protein kinase; CIPK24 (also referred to as SOS2) enables the phosphorylation of SOS1 for its activation, an event important in maintaining the Na^+^/K^+^ ratio by sustaining K^+^ transporters (Epstein, 1998; Halfter et al., 2000; Zhu, 2002).

Microbes minimize the accumulation of ions by increasing Na^+^ exclusion at the roots besides boosting the working affinity of K^+^ transporters that indirectly reduce their build-up in aerial parts, thereby contributing to the maintenance of ion homeostasis in plants. Besides promoting biofilm formation at the root surface that prevents the influx of Na^+^ into the roots, EPS production by PGPR strains traps cations in their matrix, thereby make it unavailable for uptake by the plants (Dodd and Perez-Alfocea, 2012). The inoculation of *Aeromonas hydrophila* and *Bacillus* sp. capable of producing EPS to the roots of wheat traps Na^+^ ions, thereby making it unavailable for accumulation at the leaf surface (Ashraf et al., 2004). The inoculation of *B. subtilis* GB03 to the roots of *Arabidopsis thaliana* results in the down-regulation of *HKT1*, thereby reducing the uptake of Na^+^ (Zhang et al., 2008; Qin et al., 2016). Restricting the uptake of Na^+^ at the root surface leads to induction in the expression of *HKT1* in shoots for facilitating the recirculation of Na^+^ from the shoot toward the roots, which helps in maintaining a high K^+^/Na^+^ ratio in plants (Zhang et al., 2008; Qin et al., 2016; Ali et al., 2019). With the RNA interference-mediated mutation of Ca^2+^-dependent protein kinase, CPK12 increases the sensitivity of *Arabidopsis thaliana* to salt stress (Zhang et al., 2018). The inoculation of *Azotobacter* strains C5 and C9 increases the exclusion of Na^+^ and, in anticipation, enhances K^+^ uptake, which subsequently led to an increase in proline, polyphenol, and chlorophyll content in maize leaves grown under salt stress (Rojas-Tapias et al., 2012). While studying the short- and the long-term effects of salt stress on *A. thaliana*, the inoculation of *Burkholderia phytofirmans* PsJN was found to attribute tolerance to a high amount of salts *via* alteration in the expression of ion homeostasis-associated genes (HKT1, KT1, SOS1, and Na^+^/H^+^ exchanger NHX2) (Pinedo et al., 2015). Similarly, the inoculation of *B. subtilis* GB03 to *Puccinella tenuiflora* showed an upregulation in the expression of Pt*SOS1* and Pt*HKT1* with less Na^+^ accumulation under a high salt concentration (Niu et al., 2016).

### Exopolysaccharide Production

Exopolysaccharide are homo- or hetero-polysaccharides produced by rhizobacteria that enable their survival under inhospitable conditions. Though polysaccharides vary in composition, glucose, galactose, and mannose are abundant monomers that, in association with other sub-unit components such as amino sugars, uronic acids, etc., form a capsule-like protective biofilm on the surface of cells (Upadhyay et al., 2011; Rossi and De Philippis, 2015). Formed under adverse conditions, the adsorption of EPS on soil *via* cation bridges and Van der waals forces stabilizes soil structure and aggregation (Sandhya et al., 2009). Binding soil particles to aggregates, EPS form an enclosed matrix that increases root-adhering soil per root tissue (RAS/RT), conferring protection against environmental fluctuations. The protective EPS capsule possesses strong water-holding capacity that helps in the nutrient uptake by plants besides maintaining a higher water potential around the plant roots, protecting the plant from desiccation and ensuring plant growth and survival under salinity stress (Upadhyay et al., 2011; Selvakumar et al., 2012; Balsanelli et al., 2014). In addition to its role in nodule formation in legume–rhizobia associations, it forms a protective biofilm around the roots, thereby imparting protection to plant against salinity stress (Stoodley et al., 2002; Skorupska et al., 2006). Additionally, EPS rhizosheaths around the plant roots get hold of Na^+^ ions, thereby make these unavailable to plants. The inoculation of *Halomonas variabilis* (HT1) and *P. rifietoensis* (RT4) under salinity stress stabilizes soil structures and aggregation, thereby increasing the growth of chickpea (*Cicer arietinum* var. CM-98) (Qurashi and Sabri, 2012). Exerting a capability to fight salt stress, the inoculation of *Bacillus subtilis* to *Helianthus annus* was found to downregulate the expression of HKT1/K^+^ transporter (Zhang et al., 2008). *Pseudomonas aeruginosa* inoculation reduces salt stress and promoted growth that led to an enhancement in yield in *Helianthus annus* (Tewari and Arora, 2014). EPS are also used as seed priming agents that promote seed germination and, as such, crop yield under salinity stress conditions (Tewari and Arora, 2014). The seed inoculation of *Enterobacter* sp. MN17 and *Bacillus* sp. MN54 of *Chenopodium quinoa* results in improved plant water relation following growth under a high salt (400mM NaCl) concentration (Yang et al., 2016). The inoculation of *B. subtilis* subsp. *inaquosorum* and *Marinobacter lipolyticus* SM19 significantly reduces the adverse effects of salinity stress in wheat (Atouei et al., 2019). Additionally, the inoculation of halotolerant *Pseudomonas* PS01 strain was found to be associated with the regulation of the expression of genes related to salt stress in *A. thaliana* (Chu et al., 2019).

### Production of Volatile Organic Compounds

Volatile organic compounds (VOCs; lipophilic in nature) are low molecular weight compounds that serve as signals for development and systemic response within the same or neighboring plants (Choudhary et al., 2008; Niinemets, 2010). The PGPR-mediated production of VOCs induces a range of physiological changes in plants that stimulate its growth (increasing shoot biomass) besides inducing systemic resistance to disease and controlling the plant pathogens (Lee et al., 2012; Park et al., 2015; Tahir et al., 2017). VOCs promote the biosynthesis of osmo-protectants such as glycine betaine whose accumulation imparts protection to PS-II besides maintaining the enzymatic activity and the membrane integrity of cells under osmotic stress conditions (Mäkelä et al., 2000; Jagendorf and Takabe, 2001). The VOCs of *B. subtilis* reduce salt stress through an enhancement in the tissue-specific expression of the HKT1/K^+^ transporter that enhances nutrient uptake at the root surface while minimizing the influx of Na^+^ to the roots (Zhang et al., 2008). *P. chlororaphis* O6 production of 2R, 3R-butanediol prevents water loss by inducing stomatal closures in *A. thaliana*, thereby imparting tolerance to *A. thaliana* (Cho et al., 2008). The process is mediated by Aba-1 and OST-1 kinases of jasmonic acid, ethylene, and salicylic acid pathways in plants. An increase in the VOC level on priming wheat plants with *B. thuringiensis* AZP2 imparts self-protection to the plants that enhances survival (fivefold higher) with higher photosynthesis, resulting in increased biomass under salt stress conditions (Timmusk et al., 2014). VOCs produced by *P. simiae* up-regulates γ-glutamyl hydrolase, vegetative storage (regulating Na^+^ homeostasis), and RUBISCO large-chain (associated with an increase in chlorophyll content and, as such, photosynthesis) proteins that are considered important in eliciting induced systemic resistance in soybean (*Glycine max*) (Vaishnav et al., 2015). Butanoic acid released by *Alcaligens faecalis* strain JBCS1294 attribute salt tolerance to plants *via* reprogramming of auxin and gibberellin pathways (Bhattacharyya et al., 2015). A blend of 7-hexanol, 3-methylbutanol, and 2-undecanone was found effective in mimicking VOCs in attributing plant growth effects on inoculation with different bacterial species (Ledger et al., 2016).

### Antioxidant Production

Reactive oxygen species (ROS; including superoxide O_2_**^–⋅^**, hydroxyl radical OH**^⋅^**, hydrogen peroxide H_2_O_2_, etc.), generated as a metabolic by-product in plants, functions primarily as a signaling molecule. Abnormality in the cellular metabolic process of plants growing under stress conditions enhances the production of ROS, which results in DNA damage, changes in redox state, abnormality in protein formation, denaturation of membranous proteins, lipid peroxidation, reduction in membrane fluidity, interference with enzymatic activity, and overall homeostasis of cell that progresses to cell damage and even to plant cell death (Miller et al., 2010; Halo et al., 2015). Under such conditions, both enzymatic (SOD, superoxide dismutase; CAT, catalase; APX, ascorbate peroxidase, etc.) and non-enzymatic antioxidants (GSH, glutathione; tocopherols; ascorbic acid, etc.) play a vital role in neutralizing the ROS and, as such, protect plant cells against oxidative stress (Kim et al., 2014; Kaushal and Wani, 2015). In this regard, PGPRs extend their antioxidant enzyme machinery as protection to plants against oxidative stress. Salt stress induction triggers adaptive response mechanisms, including the accumulation of compatible compounds (organic and inorganic) that decrease the hydraulic conductivity of membranes for reducing cellular osmotic stress (Hasegawa et al., 2000; Munns, 2002a; Abdul-Jaleel et al., 2007). The inoculation of *Pseudomonas* sp. to basil plants (*Ocimum basilicum* L.) grown under stress conditions results in increasing the CAT activity, while the application of a microbial consortia (*Pseudomonas* sp., *B. lentus*, and *A. brasilense*) results in enhancement in APX and GPX (Heidari and Golpayegani, 2011). Similarly, tomato seedlings inoculated with *Enterobacter* spp. showed an increase in APX activity (Sandhya et al., 2010), while the inoculation of PGPR to gladiolus showed an enhancement in SOD and CAT activities (Damodaran et al., 2013). The inoculation of PGPR to *Solanum tuberosum* grown under stress conditions results in an enhancement in the activity of APX, SOD, CAT, and glutathione reductase (Gururani et al., 2013). The inoculation of *B. amyloliquefaciens* NBRISN13 (SN13) of rice grown under salinity stress results in an enhancement in chlorophyll content and plant biomass besides increasing proline content and the expression of antioxidant enzymes such as CAT (Nautiyal et al., 2013). An up-regulation in stress-responsive genes associated with proline biosynthesis was observed on treating *A. thaliana* with *Enterobacter* sp. (Kim et al., 2014).

The inoculation of microbial consortia (*A. nitroguajacolicus* strain YB3 and YB5, *P. jessenii* R62, and *P. synxantha* R81) to IR-64 variety of rice grown under stress conditions induces and, as such, enhances SOD, peroxidase (POD), CAT, and APX levels (Gusain et al., 2015). A significant increase in the transcription of stress-responsive genes, *AtRSA1* (associated with ROS detoxification) and *AtWRKY8* (associated with maintenance of ion homeostasis), while reducing the expression of *AtVQ9* (negative regulator of *AtWRKY8*), was observed on inoculating *Paenibacillus youginensis*-to *A. thaliana* seedlings (Sukweenadhi et al., 2015). The inoculation of maize seedling with *B. amyloliquefaciens* SQR9 improved the glutathione, POD, and CAT levels besides showing an enhancement in soluble sugar and chlorophyll content (Chen et al., 2016). The physiological effects of the treatment were assessed as enhancement in *RBCL* (related to photosynthesis), *HKT1*, and *NHX-1*, -*2*, and -*3* genes. Modulation in the expression of complete gene families associated with abscisic acid (ABA) signaling, ion transport, SOS pathway, and antioxidants was observed on inoculating wheat with salt-tolerant *Dietzia natronolimnaea* (Bharti et al., 2016). The inoculation of soybean by *P. simiae* strain AU results in the enhancement of pyrroline-5-carboxylase synthase, associated with the synthesis of proline as part of tolerance to stress conditions (Vaishnav and Choudhary, 2019). The study goes well with previous reports regarding the enhancement in proline content during stress conditions (Ghosh et al., 2018; Patel et al., 2018). The inoculation of *Azospirillum lipoferum* FK1 of chickpea exhibited enhanced antioxidant enzyme levels besides demonstrating an increase in nutrient uptake and, as such, improvement in its growth and development (El-Esawi et al., 2019). The bacterial consortium of *P. fluorescens* S3, *B. mojavensis* S1, and *B. pumilis* mitigates salt-induced growth inhibition of barley through an enhancement in the water conductance and the nutrient uptake of plants. The inoculation of rice with *Trichoderma asperellum* and *P. fluorescens* results in an enhancement in the activity of POD, APX, SOD, and CAT that contributes to the alleviation of salt stress (Singh et al., 2020).

### Enzymes and Metabolites of Bacterial Origin

Plant diseases are considered as a major constraint to crop yield. It has been observed that salinity stress contributes to an increase in the susceptibility of plants to attacks by different pathogens (Besri, 1993). As the usage of chemicals in the control of plant pathogens imparts deleterious effects, PGPR emerged as a potential substitute as a biological control strategy in the management of pathogen-associated diseases in plants (Compant et al., 2010; Etesami and Alikhani, 2018). PGPR-based mechanisms employed in the biological control of pathogens include the following:

(1) Synthesis of cell-wall-degrading enzymes: The production of hydrolytic enzymes such as cellulases, glucanases, chitinases, protease, etc., hydrolyzing polymeric compounds such as cellulose, hemicellulose, chitin, cell wall proteins, etc., was found capable of inhibiting a variety of plant pathogens (Pal and Gardener, 2006; Mabood et al., 2014; Husson et al., 2017; Vaddepalli et al., 2017). Similarly, protease produced by different PGPR agents was found effective in reducing the infections of *Fusarium* sp. and *M. phaseolina* (Dunne et al., 1997; Gohel et al., 2004). The biocontrol potential of chitinase produced by *Paenibacillus illinoissensis* spp. provides protection against blight and damping off diseases in pepper (*Capsicum annuum*) caused by *Phytophthora capsica* and *Rhizoctonia solani* (Jung et al., 2003, 2005). Chitinase produced by *B. suly* reduces the infection severity of *Fusarium* sp. under greenhouse conditions (Hariprasad et al., 2011). The production of chitinases together with β-1,3-glucanases by PGPR such as *B. subtilis* BSK17 for utilizing them as a source of carbon is of prime importance as it forms a major enzyme group capable of degrading the chitin and laminarin components of fungal cell walls (Kumar et al., 2012; Dubey et al., 2014).
(2) Synthesis of antimicrobial metabolites: With maximum reports from *Bacillus* and *Pseudomonas* genera, the production of a wide range of metabolites was found to restrict the growth of pathogens (Couillerot et al., 2009; Olanrewaju et al., 2017).
(3) HCN production by *Pseudomonas* sp., *Bacillus* sp., *Rhizobium*, etc., was found capable of inhibiting cytochrome C oxidase along with other metalloenzymes (Nandi et al., 2017).
(4) The synthesis of siderophores by different PGPR strains possessing a high affinity for Fe^3+^ ions chelates it and, as such, deprives pathogens of this essential mineral (Shen et al., 2013; Olanrewaju et al., 2017).
(5) Prime plants for induction of induced systemic resistance that imparts a faster and stronger response to attacks by different pathogens (Olanrewaju et al., 2017).

### Maintenance of Hormonal Balance

Phytohormones regulating plant growth and developmental processes attributes plants protection by imparting tolerance to cope up with diverse changes in the environment (Ryu and Cho, 2015). The exogenous application of phytohormones supplementing the internal hormonal pool was found effective in counteracting the deleterious effects of salt stress (Zahir et al., 2010). The exogenous application of indole-3-acetic acid (IAA) was found effective in stimulating the growth of roots and leaves, thereby alleviating salinity-induced reduction in plant productivity (Albacete et al., 2008; Dodd and Perez-Alfocea, 2012). Diminishing the endogenous hormonal level, metabolites, hormones, and enzymes produced by salt-tolerant (ST) PGPR complements the hormonal status of plants and, as such, contributes to the enhancement of salt tolerance in plants grown under salt stress (Egamberdieva and Kucharova, 2009; Ilangumaran and Smith, 2017). A common trait of PGPR, production of IAA, was found to increase the fitness of plants grown under salinity stress (Dodd et al., 2010; Tiwari et al., 2011). Tryptophan in root exudates is utilized by rhizobacteria for its conversion through multiple routes to IAA for it to be readily absorbed by plant roots (Spaepen and Vanderleyden, 2011; Ilangumaran and Smith, 2017). Complementing the endogenous IAA pool of plants, its function in plants depends on the internal IAA levels (ranging in function from promotion to inhibition of plant growth). Required for cell division and elongation in plants, the inoculation of ST-PGPR *P. putida* modulated internal IAA pools that resulted in an increase in the growth parameters in cotton plants grown under salinity stress (Yao et al., 2010; Egamberdieva et al., 2017). The inoculation of *P. stutzeri*, *P. putida*, and *Stenotrophomonas maltophilia* to *Coleus* plants was found to lead the production of IAA, cytokinin, and gibberellic acid (Patel and Saraf, 2017). The short-term treatment of *Enterobacter* sp. EJ01 increased the expression of salt stress-responsive genes such as late embryogenesis abundant (*RAB18*), DRE-binding protein (*DREB2b*), stress-inducible priming process (*MPK3* and *MPK6*), etc., genes in *Arabidopsis thaliana*, while increasing the ROS scavenging activity of *Solanum lycopersicum* grown under salinity stress (Ilangumaran and Smith, 2017). The inoculation of halotolerants was found to be associated with an increase in the secretion of salicylic acid that leads to an enhancement in the growth of sunflower plant (Tewari and Arora, 2018). The inoculation of *Leclerciaa decarboxylata* MO1 in *Solanum lycopersicum* showed an improvement in chlorophyll fluorescence besides increasing sugar synthesis and the production of organic acids (Kang et al., 2019).

Cytokinin (CK) is another important class of phytohormones that assists plants in growth and development and in attributing resistance to different stresses (O’Brien and Benková, 2013). Though a common trait of PGPRs, they suffice plants of CK by either synthesizing it or altering its homeostasis in plants (Dodd et al., 2010; Pallai et al., 2012; Kapoor and Kaur, 2016). The inoculation of *Pseudomonas* sp. (*P. aurantiaca* and *P. extremorientalis* TSAU6 and TSAU20) results in alleviating the salinity-induced dormancy of wheat seeds besides enhancing their growth under salinity stress conditions (Egamberdieva, 2009). The inoculation of *B. subtilis* strain in *Platycladus orientalis* and lettuce plant showed an enhanced root-to-shoot signaling of CK, thereby improving plant growth under stress conditions (Arkhipova et al., 2007; Liu et al., 2013). The ability of PGPRs to synthesize CK highlights their importance in stimulating plant growth.

Gibberellins (GA) constitute another important class of phytohormones that play an important role in regulating cell division and elongation and in regulating meristematic activity at the roots and the leaves as part of its role in the developmental and physiological processes of plants (Wang et al., 2015; Guo et al., 2015; Martínez et al., 2016). Bottini et al. reported the production of gibberellin by PGPR strains of *B. licheniformis*, *B. pumilis*, and *Azospirillium* spp. (Bottini et al., 2004). Being a key factor associated with the inhibition of plant growth under stress conditions, PGPRs were found to enhance its levels in plants, thereby attributing a tolerance mechanism to plants for growth under salinity stress (Kang et al., 2014a; Martínez et al., 2016; Shahzad et al., 2016). Kang et al. (2014a) reported enhancement in the internal GA pools on inoculating plants with *B. cereus* MJ-1 and *Promicromospora* sp. SE188. A similar effect of regulating plant growth and development was observed on inoculating plants with *B. aryabhattai* SRB02 (Park et al., 2017). The inoculation of *P. aeruginosa* PM389 and ZNP1 together with *B. endophyticus* J13 and *B. tequilensis* J12 results in the alleviation of the stress-induced effects in *A. thaliana* (Ghosh et al., 2019).

Abscisic acid is a stress hormone primarily known for its role in the abscission of leaves and growth of plants. Synthesized under water deficit conditions, it triggers an adaptive response *via* the activation of a set of genes responsible for stress resistance as part of its survival strategy for the plants (Pliego et al., 2011; Sah et al., 2016). Its synthesis in the roots that occurs in response to low water potential triggers the growth of roots and the emergence of lateral roots, contributing to the enhancement in the uptake of water at the root surface (Vaishnav et al., 2016b). Simultaneously, its translocation from roots to leaves progresses with the control of the stomatal closure events toward regulation of water loss by reducing transpiration at the leaf surface (Yamaguchi-Shinozaki and Shinozaki, 1994; Dodd and Perez-Alfocea, 2012; Kaushal and Wani, 2015). PGPRs capable of producing ABA play an important role in plant–PGPR interactions (Dodd, 2003; Naz et al., 2009; Dodd et al., 2010). They either modulate the biosynthesis of ABA or regulate ABA-mediated signaling pathways in plants, thereby contributing to the growth and survival of plants under salinity stress. The inoculation of PGPRs often mitigate the sensitivity of plants to water scarcity by decreasing its accumulation at the roots and significantly altering its long-distance signaling, i.e., shoot-to-root or *vice versa* flow through the phloem and the xylem, respectively (Dodd and Perez-Alfocea, 2012; Jiang et al., 2012; Belimov et al., 2014). The inoculation of *Phyllobacterium brassicacearum* STM196 results in an enhancement of the ABA levels that reduces transpiration at the leaf surface and, as such, enhances salt stress tolerance in *A. thaliana* (Bresson et al., 2013). A few species of PGPR (*Rhodococcus* sp. and *Novosphingobium* sp.) inhabiting rhizospheric regions capable of metabolizing ABA under *in vitro* conditions represent another stress-relieving mechanism for plants (Belimov et al., 2014). The inoculation of plants with ABA-producing PGPRs (*P. fluorescence* Rt6M10, *A. brasilense* SP245, and *B. licheniformis* Rt4M10) results in enhancement in internal ABA pools, thereby increasing plant growth under salinity stress conditions (Salomon et al., 2014; Cohen et al., 2015). A study reported that PGPR stimulated the production of endogenous ABA in plants, relieving them of the effects of being grown under salinity stress (Forni et al., 2017). Both ABA synthesizing and metabolizing PGPRs are capable of modulating the internal ABA status of plants and, as such, are capable of relieving plants to show normal growth even under salinity stress conditions.

Apart from ABA, the synthesis of another stress hormone, ethylene, was found to improve tolerance or expedite senescence (Morgan and Drew, 1997). Ethylene, a gaseous hormone, significantly enhances the response of plants to stress conditions. Acting as a negative regulator of plant growth, ethylene induces its effects by reducing the growth of roots and modulating the nitrogen-fixing capability of plants (Ma et al., 2002; Mahajan and Tuteja, 2005; Gamalero and Glick, 2015). As ethylene-mediated inhibition of the auxin response factor constraints the growth of plants, secretion of 1-aminocyclopropane-1-carboxylase (ACC) deaminase by PGPR hampers its synthesis in plants (Glick et al., 2007). ACC deaminase secretion by PGPR metabolizes ACC (precursor of ethylene in plants) into α-ketoglutarate and ammonia besides altering the expression of genes encoding ACC synthase and ACC oxidase, which are involved in the synthesis of ethylene (Etesami and Beattie, 2017). ACC deaminase-producing strains of *P. fluorescens* and *Enterobacter* spp. produced a significant effect in increasing the yield of maize grown under salt stress conditions (Nadeem et al., 2009; Panwar et al., 2016). The inoculation of *Pantoea dispera* PSB3 to chickpea results in an enhancement in IAA and ACC deaminase production, which led to an improvement in pod size, seed weight, seed number, and altogether plant biomass (Panwar et al., 2016). The plants were also observed to have a higher K^+^/Na^+^ ratio, owing to a reduction in electrolyte leakage and a decreased uptake of Na^+^ besides leading to an increase in leaf water and chlorophyll content and enhancement in K^+^ uptake.

## Conclusion and Future Perspectives

Though much progress has been made in understanding the different attributes of plant–microbe interactions and in formulating methodologies for crops grown under salinity stress, we still lag behind in achieving sustainability in plant productivity. With rising emphasis on environmental protection and sustainability in agriculture for food security, the timely mitigation of the adverse effects of different stresses, in a cost-effective manner, is required. For this to be realized, it becomes imperative to explore novel aspects of the plant-beneficial soil microbiota in relieving plants of stressful conditions. Microbiota from diversified environments needs characterization and exploration in terms of their acclimatization, in-depth knowledge of their ameliorative strategies for growth under stress conditions, and in acquiring knowledge of the intriguing mechanisms commonly employed in attributing plants with a potential to thrive in harsh edaphic conditions. As the efficiency of the microbiota depends on soil characteristics and plant species, a better understanding of plant–microbial interactions in the context of manipulation of stress-responsive genes in plants need further elucidation in terms of revealing their functionalities toward boosting plant defense and attaining enhancement in overall productivity. As the soil microbiota provides beneficial attributes to plants in withstanding salinity stress, newer prospects of understanding in the operational module of regulatory network-mediated plant defense in achieving tolerance against different stresses need to be undertaken in a timely manner. The same goes in terms of prospects of developing novel bioinoculants that could enhance the stability of crops grown under stress conditions and, as such, increase their productivity when grown in nutritionally poor agroecosystems. In addition to the screening and the optimization of PGPR strains for plant-beneficial characteristics under changing environmental conditions, the CRISPR/Cas approach in editing interactive networks of stress-responsive genes needs to be undertaken for their profound effect (metabolic, regulatory, and signaling) in overcoming stress and inducing tolerance in plants and their interacting partners toward attaining sustainability in agriculture production.

## Author Contributions

AJ and SR conceived the idea. All authors contributed equally in generating the draft of the different sections and in the repeated editing of the contents present in the finalized version of the manuscript.

## Conflict of Interest

The authors declare that the research was conducted in the absence of any commercial or financial relationships that could be construed as a potential conflict of interest.
